# Effects of Enzymatic Pretreatment of Seeds on the Physicochemical Properties, Bioactive Compounds, and Antioxidant Activity of Pomegranate Seed Oil

**DOI:** 10.3390/molecules26154575

**Published:** 2021-07-28

**Authors:** Tafadzwa Kaseke, Umezuruike Linus Opara, Olaniyi Amos Fawole

**Affiliations:** 1 Department of Food Science, Faculty of AgriSciences, Stellenbosch University, Private Bag X1, Stellenbosch 7602, South Africa; tafakaseqe@gmail.com; 2 SARChI Postharvest Technology Research Laboratory, Africa Institute for Postharvest Technology, Faculty of AgriSciences, Stellenbosch University, Stellenbosch 7600, South Africa; 3 UNESCO International Centre for Biotechnology, Nsukka 410001, Enugu State, Nigeria; 4 Department of Botany and Plant Biotechnology, Faculty of Science, University of Johannesburg, P.O. Box 524, Johannesburg 2006, South Africa

**Keywords:** pomegranate seed, oil, seed enzymatic pretreatment, yellowness index, total phenolic content, phytosterols, punicic acid, antioxidant capacity

## Abstract

Enzymatic pretreatment of seeds is a novel approach that enhances the health benefits of the extracted oil. The study investigated the influence of the enzymatic pretreatment of seeds on the quality of oil from different pomegranate cultivars. The quality of the ultrasound-assisted (and ethanol-extracted) oil was studied, with respect to the refractive index (RI), yellowness index (YI), conjugated dienes (K232), peroxide value (PV) ρ-anisidine value (AV), total oxidation value (TOTOX), total carotenoid content (TCC), total phenolic compounds (TPC), fatty acid composition, phytosterol composition, ferric reducing antioxidant power (FRAP), and 2.2-diphenyl-1-picryl hydrazyl (DPPH) radical scavenging capacity. The seeds of three different pomegranate cultivars (‘Wonderful’, ‘Herskawitz’, and ‘Acco’) were digested with an equal mixture of Pectinex Ultra SPL, Flavourzyme 100 L, and cellulase crude enzymes, at a concentration, pH, temperature, and time of 1.7%, 4.5, 40 °C, and 5 h, respectively. Enzymatic pretreatment of PS increased oil yield, PV, TPC, TCC, and DPPH radical scavenging capacity, but decreased the YI. The levels of K232, AV and TOTOX, fatty acids, phytosterols, RI, and FRAP, were not significantly affected by enzymatic pretreatment of PS. Principal component analysis (PCA) established that oil extracted from the ‘Acco’ seed after enzymatic pretreatment had higher yield, TPC, TCC, and DPPH radical scavenging capacity. Therefore, enzyme-pretreated ‘Acco’ pomegranate fruit seed is a source of quality seed oil with excellent antioxidant properties.

## 1. Introduction

The pomegranate (*Punica granatum* L.) seed, a by-product of pomegranate fruit processed into juice and other products, is a rich source of pharmaceutical and nutraceutical compounds, such as tocopherols, sterols, carotenoids, polyphenols, and punicic acid [1,2]. Punicic acid, a conjugated polyunsaturated fatty acid, is an essential fatty acid that is primarily obtained from pomegranate seeds (PS) [3]. The omega-5 fatty acid possesses a wide range of health benefits, including antidiabetic, antiobesity, antiproliferative, anticarcinogenic, nephroprotective, and neuroprotective activities [4,5]. Pomegranate seed oil (PSO), with its diverse biological properties, could be utilised in the formulation of functional foods and nutraceutical products. Despite this, PS are still largely considered as waste, further increasing the total pomegranate fruit postharvest losses [6]. Processing PS into specialty oil would not only add value to the waste from pomegranate fruit processing, but also contributes to postharvest waste management solutions [3].

Conventionally, the seed oil is extracted using mechanical pressing or organic solvents. Despite producing oil rich in bioactive compounds, mechanical pressing is associated with low oil yield and high energy consumption [7]. Extraction of seed oil with organic solvents, such as hexane, presents a higher oil extraction rate, and has been used for decades in conjunction with mechanical pressing. However, there are increasing consumer concerns over the continued use of hexane in food extraction due to its detrimental effects on humans and the environment [8]. Furthermore, hexane is a strong non-polar solvent and has low affinity for polar bioactive compounds, such as phenols; therefore, it would not be the best solvent to use in the production of specialty oils, such as PSO [9]. Therefore, researchers are increasingly focusing on alternative solvents, which are generally regarded as safe (GRAS), and could produce high quality oil.

Among low chain alcohol-based organic solvents, ethanol is regarded as safe for seed oil extraction and is authorized for food extraction by the European Directive 2009/32/EC [10]. Moreover, ethanol is cheaper, readily available, and bio-renewable [11]. The main setback with the use of ethanol in seed oil extraction is that it produces low oil yield [12]. Alteration of the oilseed microstructure prior to oil extraction may enhance ethanol seed oil extraction efficiency. Enzymatic pretreatment of oilseeds has emerged as a novel and effective method to enhance the recovery of oil and bioactive compounds without degrading the oil quality. Enzyme digestion increases the permeability of the cell walls to the intracellular material by breaking down the complex polysaccharides and lipoproteins into simple molecules [13]. This reduces the oil extraction time, enzyme, solvent, and energy consumption [14].

Previous studies have reported improved oil yield and bioactive compound recovery from various oilseeds by enzymatic pretreatment [15,16,17]. The use of enzymes in oilseed pretreatment depends on the seed composition. Seed cells are surrounded by a complex cell wall composed of polysaccharides (cellulose, hemicellulose, and pectin) and protein [15,18]. Seed oil bodies are surrounded by phospholipids and a protein membrane [19]. Application of enzyme concentrations, with a wide range of activities is therefore vital for maximum recovery of oil and bioactive compounds. Passos et al. [15] applied a combination of cellulase, hemicellulase, protease, and pectinase enzymes to extract grape seed oil, and reported a 192% increase in oil yield. Pretreatment of borage seed with an equal mixture of Olivex and Celluclast significantly enhanced the methanol oil extract’s total phenolic composition by 61% [20].

Due to differences in genetic characteristics, the concentration of polysaccharides, proteins, and lignin may vary among cultivars and, therefore, the efficiency of enzymes in hydrolysing the linkages between the lipids, carbohydrates, and bioactive compounds may also be cultivar dependent [13]. From economic and industrial viewpoints, plant cultivars with the most desirable seed oil quality attributes, after pre-treating seeds with enzymes, are valuable to oil processors, manufacturers of food and nutraceuticals, and consumers. With increasing consumer interest and demand for pomegranate seed oil, there is interest among fruit growers and processors on the selection of cultivars for better returns from oil processing. However, information on enzymatic pretreatment of seeds to enhance oil yield and the quality of different fruit cultivars is scarce in the literature. Therefore, in this study, we pre-treated pomegranate seeds with enzymes, with the objective to investigate the effects on the quality and antioxidant capacity of oil from three different cultivars.

## 2. Results and Discussion

### 2.1. Oil Yield and Seed Microstructure Changes

**Figure 1:**
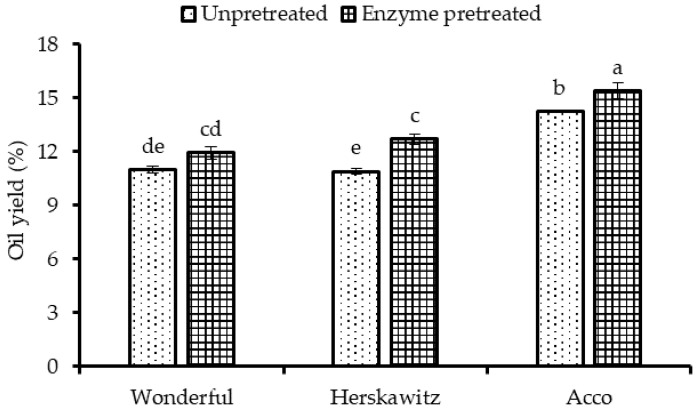
Oil yield from un-pretreated and enzyme pretreated seeds (1.7%, 40 °C, pH = 4.5 and 5 h) of three pomegranate cultivars. Within the same cultivar (un-pretreated and enzyme pretreated), columns followed by different letters are significantly different (*p* < 0.05) according to Duncan’s multiple range test. Vertical bars indicate the standard deviation of the mean.

**Figure 2:**
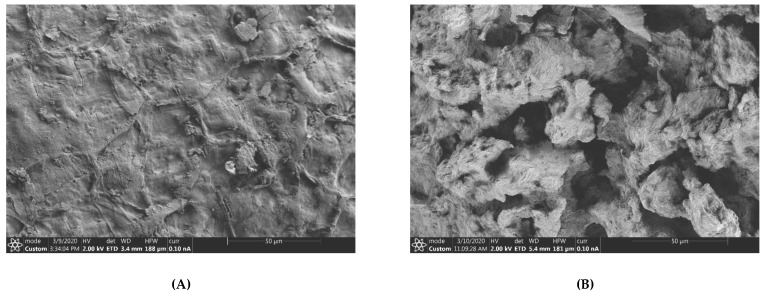
Representative SEM micrographs showing the effect of enzymatic pretreatment (1.7%, 40 °C, pH = 4.5 and 5 h) on the pomegranate seed microstructure (**A**) un-pretreated pomegranate seed, (**B**) enzyme pretreated pomegranate seed.

The PSO yield, which ranged from 10.90 to 15.40%, varied significantly among the different pomegranate cultivars (Figure 1). In addition, pre-treating PS with enzymes significantly improved ‘Acco’ and ‘Herskawitz’ oil yield by 8 and 17%, respectively. On the other hand, no significant (*p* > 0.05) increase in oil yield was observed on ‘Wonderful’ after seed enzymatic pretreatment. As shown in Figure 1, ‘Acco’ (15.40%) (dw) exhibited significantly higher oil yield than ‘Herskawitz’ (12.70%) (dw) and ‘Wonderful’ (11.93%) (dw) after PS enzymatic pretreatment, an indication that the cultivars responded differently to enzymatic pretreatment. According to Sarkhosh et al. [21], this phenomenon can be explained by the cultivar’s variation in genetic characteristics. In line with this, the amount and type of PS cellulose, hemicellulose, pectin, and protein may also significantly vary among the cultivars, influencing the PS digestibility by enzymes. The significant effects of seed enzymatic pretreatment on oil yield was reported in prior research. For instance, Passos et al. [15] and Li et al. [22] reported a remarkable increase in oil yield after pre-treating grape and Silybum marianum seed, respectively, with a cocktail of enzymes. The effect of enzymatic pretreatment of seed microstructure was evaluated by scanning electron microscopy (SEM) on un-pretreated and enzyme-pretreated PS powders. Figure 2a shows am SEM micrograph from un-pretreated PS, characterised by visible and intact cell walls. After enzymatic pretreatment, the seed matrix was extensively raptured (Figure 2b). In addition, void spaces were observed in the SEM micrograph from enzyme pretreated seeds, suggesting that the seed cell walls and oil bodies were disintegrated after enzymatic pretreatment, thereby facilitating mass transfer of lipids into the extraction solvent [23]. This could explain the significant increase in PSO yield from ‘Acco’ and ‘Herskawitz’ oil extracts after enzymatic pretreatment (Figure 1). In a previous study, SEM analysis of Silybum marianum seed powders also confirmed significant alteration of the seed cells and oil yield improvement after enzymatic pretreatment [22]. In the seed cells, oil exists in the form of oil bodies (0.5–2.5 µm) that are surrounded by a protein membrane, which could also be hydrolysed by the exogenous protease enzymes [19].

### 2.2. Refractive and Yellowness Index

In freshly processed seed oil, refractive index (RI) may be directly related to molecular weight, degree of unsaturation, and degree of fatty acids conjugation. Therefore, RI may be used as an indirect way of assessing the oil stability and susceptibility to oxidation. The results in Table 1 show that the oil RI did not significantly (*p* > 0.05) vary after PS enzymatic pretreatment and among the cultivars, suggesting that enzymatic pretreatment of PS did not significantly cause oil quality degradation (Table 1). The RI values from the current study (1.5175 to 1.5177) are comparable to those reported by Costa et al. [24] (1.5091–1.5177) from cold pressed PSO, but higher than the RI values reported from other fruit seed oils, such as grape (1.445 to 1.468) and avocado seed oil (1.3950–1.4335), indicating that PSO is higher in polyunsaturated fatty acids [25,26].

**Figure 3:**
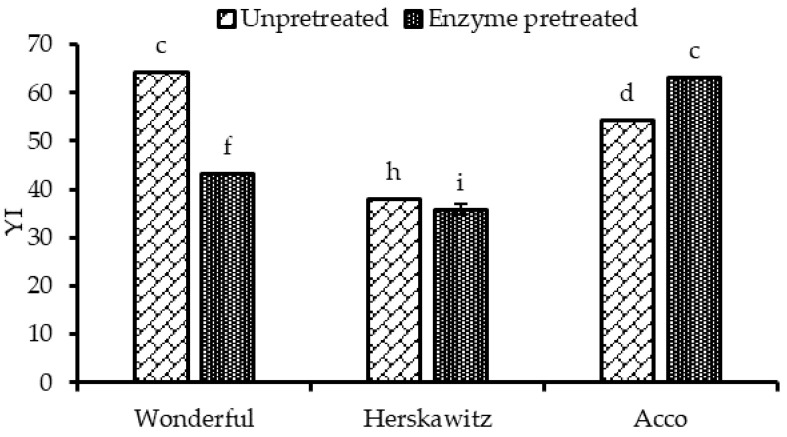
The yellowness index (YI) of un-pretreated and enzyme pretreated seeds (1.7%, 40 °C, pH = 4.5 and 5 h) from studied pomegranate cultivars. Within the same cultivar (un-pretreated and enzyme pretreated), columns followed by different letters are significantly different (*p* < 0.05), according to Duncan’s multiple range test. Vertical bars indicate the standard deviation of the mean.

Colour is the most important physical attribute of food; therefore, it holds a preeminent position in the customer’s perception of the overall food quality [27]. Seed oil colour may be influenced by the chemical changes that occurs during processing, including seed enzymatic pretreatment. The results in Figure 3 indicate that PS enzymatic pretreatment significantly decreased the yellowness index (YI) of oil extracted from ‘Herskawitz’ and ‘Wonderful’ by 5 and 38%, respectively, despite the significant increase in total carotenoid content after PS enzymatic pretreatment, particularly in the oil extracted from ‘Herskawitz’. This may be partially explained by non-enzymatic oxidation of the phenolic compounds during seed oil processing [28]. On the other hand, the YI of ‘Acco’ oil extracts significantly increased by 16% after PS enzymatic pretreatment; this could be attributed to the increased extractability of colour pigments, such as carotenes and xanthophylls, facilitated by the extensive rapture of the seed matrices (Figure 2). The difference in the cultivars’ genetic material could explain the variation in the extractability of the carotenoid compounds. The extensive conjugated double bond system in carotenoids absorbs light in the visible region and provides carotenoids with the ability to colour foods such as seed oil [29]. Improvement of oil colour after seed enzymatic pretreatment was reported in the literature. For instance, Latif and Anwar [16] reported that oil yellowness significantly improved between 25 and 27% after hempseed enzymatic pretreatment.

### 2.3. Peroxide Value, Conjugated Dienes, ρ-Anisidine Value, and Total Oxidation Value

The results in Table 1 show that low oil peroxide values (0.04–0.33 meqO_2_/kg PSO) were obtained in all of the cultivars, despite significantly increasing by 50% in ‘Wonderful’ and ‘Acco’ oil extracts after PS enzymatic pretreatment. Among the cultivars, ‘Acco’ oil extracts exhibited the highest peroxide value (PV) after PS enzymatic pretreatment, despite the increased carotenoids and phenolic compounds extraction. As shown in Figure 1, ‘Acco’ exhibited the highest oil yield after PS enzymatic pretreatment; therefore, it can be hypothesized that more damage occurred in the seed matrices, thereby increasing the oil surface area exposure to hydrolytic oxidation. According to the Codex Alimentarius Commission standard (CODEX STAN 19-1981) [30], the maximum permissible levels of PV in unrefined seed oil is 15 meqO_2_/kg oil. For PV, the PSO quality in the present study conforms to the standard and, thus, is acceptable on the international market. In a previous study, Basiri [31] also reported low PV (0.79 meqO_2_/kg PSO) from petroleum ether PSO extracts, which suggested that PSO extracts significantly resist oxidation during processing, which can be ascribed to the high levels of antioxidant compounds. 

The formation of hydroperoxides coincides with the generation of fatty acids with conjugated double bonds, commonly referred to as conjugated dienes (K232) [32]. As indicated in Table 1, pretreatment of PS with enzymes did not significantly affect the oil K232 values, although ‘Wonderful’ (0.28) oil extracts exhibited higher K232 values than ‘Herskawitz’ (0.20) and ‘Acco’ (0.17) oil extracts after pretreatment of PS with enzymes. In addition to low temperatures applied during PS enzymatic pretreatment, freeze drying the hydrolysed seed could also have minimized fatty acid oxidation. Previously, the study of Latif and Anwar [16] also reported an insignificant effect of hempseed enzymatic pretreatment on the K232 values of extracted oil.

The primary products of fatty acids oxidation are unstable and, therefore, can be further degraded to secondary products, such as the carbonyl compounds. Due to their low threshold values, and a major contribution to the development of rancid taste and flavour in seed oil, aldehydes are considered important compounds for the evaluation of secondary oxidation products in seed oil. The ρ-anisidine value (AV) measures the generation of aldehydes, primarily the 2-alkenals and 2.4-alkadienals from the decomposition of the hydroperoxides [32]. The effect of PS enzymatic pretreatment on the extracted oil AV is presented in Table 1. Whilst the oil extracted from ‘Wonderful’ exhibited a 34% decrease in AV after PS enzymatic pretreatment, ‘Herskawitz’ and ‘Acco’ oil extracts did not show a significant change in AV after seed enzymatic pretreatment. The results suggest that there was a minimum further degradation of hydroperoxides to form secondary products, due to the antioxidant effect of the carotenoids and phenolic compounds [33].

Total oxidation (TOTOX) value is used to estimate the overall oxidative degradation of fatty acids through the summation of PV and AV in fats and oils. It is clear in Table 1 that PS enzymatic pretreatment did not significantly affect the oil TOTOX value, signifying that the phenolic and carotenoid compounds either prevented the initiation of hydroperoxides generation or scavenged the intermediate products of hydroperoxides formation, such as the peroxyl radicals, thereby inhibiting the propagation of radical chain reactions during PSO processing [34,35].

### 2.4. Total Carotenoid Content, Total Phenolic Content, and Antioxidant Capacity

**Figure 4:**
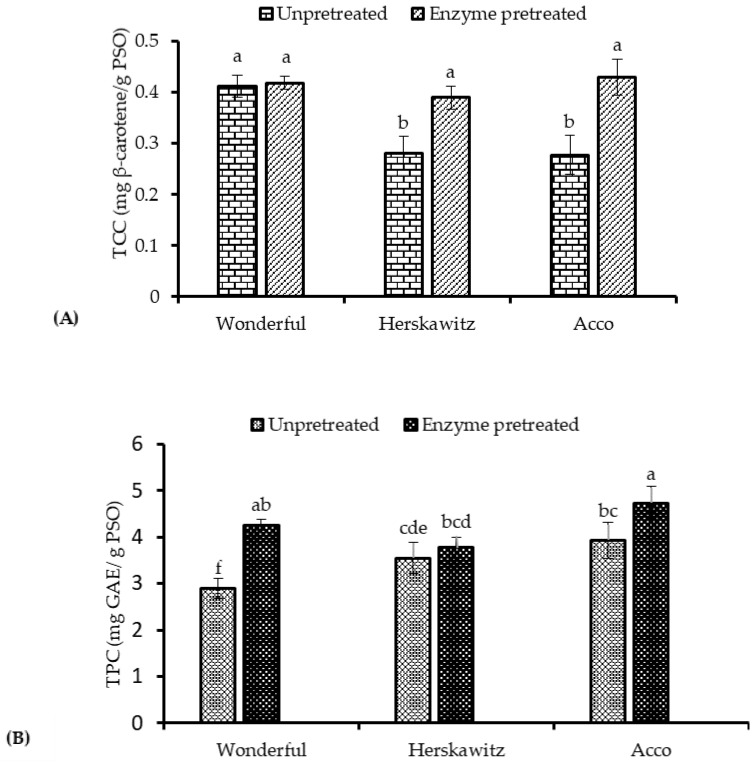
(**A**) Total carotenoid content (TCC) and (**B**) total phenolic compounds content (TPC) of un-pretreated and enzyme pretreated seeds (1.7%, 40 °C, pH = 4.5 and 5 h) from three pomegranate cultivars. Within the same cultivar (un-pretreated and enzyme pretreated), columns followed by different letters are significantly different (*p* < 0.05), according to Duncan’s multiple range test. Vertical bars indicate the standard deviation of the mean.

Apart from being natural food colour pigments, carotenoids possess biological properties that include antioxidant activity [36]. In line with this, enhancement of carotenoids extraction from oilseeds is essential to preserve the oil quality naturally. Treating PS with enzymes before oil extraction significantly enhanced the total carotenoid content (TCC) of ‘Herskawitz’ and ‘Acco’ oil extracts by 1.4 and 1.5-fold, respectively, whilst it did not significantly improve the TCC of oil extracted from ‘Wonderful’. The results indicate that cultivar significantly influenced the enzyme hydrolysis of the carotenoprotein complexes and the mass transfer of carotenoids from the seed cells into an extraction solvent [29]. As can be seen in Figure 2b, the extensive damage on the seed cells after enzymatic pretreatment, enhanced the extractability of the lipids and carotenoids (Figure 1 and Figure 4a). According to Szabo et al. [37], lipids are crucial in the extraction of carotenoids from the food matrix and absorption in the human body as they facilitate micelle production through biliary secretion, which enhances carotenoid absorption rate. Cultivar difference was reported to influence the extraction of TCC from the seed matrix significantly. For example, Gornas et al. [38], De Santana et al. [39], and Zhang et al. [40] reported a significant variation in TCC among sour cherry, passion, and black tartary buckwheat cultivars, respectively. According to Figure 4a, the pomegranate seed oil TCC from the current study ranged from 0.28 to 0.43 mg β-carotene/g PSO and was 30–53-fold higher than the TCC reported by Costa et al. [24] from cold pressed pomegranate seed oil. The dissimilarities in TCC of oil from the two studies could be ascribed to cultivar, seed oil extraction methods, and fruit growing region differences among other factors. 

Phenolic compounds have been linked with the reduction of certain chronic diseases, including cancer and cardiovascular diseases [41]. According to Kumar et al. [42], phenolic compounds more often exist as esters or glycosides than as free molecules, and therefore pretreatment of the oilseeds to break the hydrogen and covalent bonds and dissociate the polyphenols from the complex compounds is vital. As shown in Figure 4b, the total phenolic compounds (TPC) significantly increased in ‘Acco’ and ‘Wonderful’ oil extracts by 1.2- and 1.5-fold, respectively, after PS enzymatic pretreatment. It can be explained that pretreatment of PS with enzymes hydrolysed the linkages between the phenolic compounds, lipoproteins and lipopolysaccharides generating more free phenolic compounds, which were solubilized by the extraction solvent. In a similar study by Soto et al. [20], enzymatic pretreatment of borage seed significantly enhanced the methanol oil extracts total phenolic compounds by 65%. Although Figure 2b shows that there was extensive damage of the PS after enzymatic pretreatment, the concentration of TPC in ‘Herskawitz’ oil extracts did not significantly increase after pre-treating the PS with enzymes, demonstrating the significance of cultivar in the recovery of TPC from enzyme pretreated PS. Previous studies have also reported similar findings. For instance, Jing et al. [43] and Xi et al. [44] established significant variation in phenolic compounds of seed oil extracted from different pomegranate and lemon cultivars, respectively. The pomegranate seed oil TPC ranged from 2.89 to 4.73 mg GAE/g PSO. These values were lower than the TPC values reported by Abbasi et al. [45] from hexane (8.2–9.0 mg/g PSO) and supercritical carbon dioxide (7.8–72.1 mg/g PSO) extracted PSO. Several factors, including the seed oil extraction techniques, could explain the difference in TPC values.

**Figure 5:**
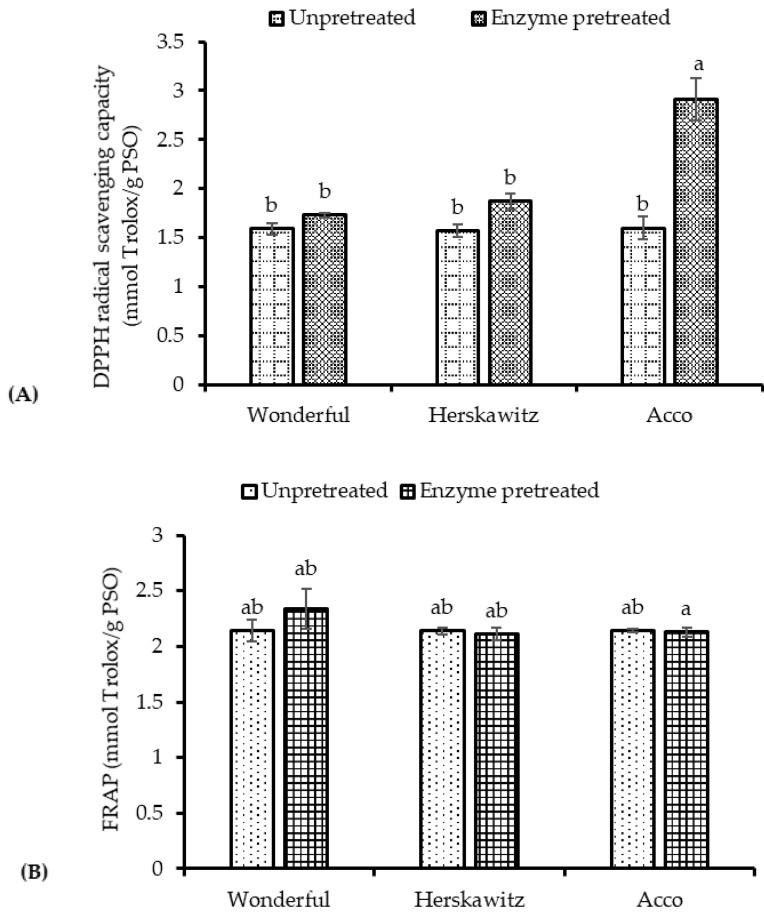
(**A**) DPPH radical scavenging capacity and (**B**) ferric reducing antioxidant power (FRAP) of un-pretreated and enzyme pretreated seeds (1.7%, 40 °C, pH = 4.5 and 5 h) from studied pomegranate cultivars. Within the same cultivar (un-pretreated and enzyme pretreated), columns followed by different letters are significantly different (*p* < 0.05), according to Duncan’s multiple range test. Vertical bars indicate the standard deviation of the mean.

Natural antioxidants may function as reducing agents, free radical scavengers, complexers of pro-oxidant metals or quenchers of the singlet oxygen [46]. In the present study, PSO antioxidant capacity was evaluated through its ability to scavenge DPPH radicals and reduce the ferric tripyridyl triazine (Fe^3+^-TPTZ) to the ferrous (Fe^2+^) state. PS enzymatic pretreatment significantly increased the DPPH radical scavenging capacity of ‘Acco’ oil extracts from 1.60 to 2.91 mmol Trolox/g PSO, but did not significantly increase the DPPH radical scavenging capacity of ‘Wonderful’ and ‘Herskawitz’ oil extracts, in spite of the significant increase in TPC and TCC (Figure 5a,b). The findings suggest that ‘Acco’ is a desirable cultivar for the recovery of bioactive compounds and enhancement of antioxidant properties with the enzymatic pretreatment. Pre-treating PS with enzymes did not significantly change the ferric reducing antioxidant power (FRAP) of oil extracts from all cultivars, and this revealed that the antioxidant assays differ in their mode of action and may influence the experimental results [43]. The findings are comparable to the results from Pande and Akoh [47], who reported a lack of variation in FRAP of hydrophilic and hydrophobic extracts from the seeds of six different pomegranate cultivars

### 2.5. Phytosterols Composition

The effect of PS enzymatic pretreatment on phytosterol composition is presented in Table 2. Two different phytosterols, including β-sitosterol and stigmasterol were identified and quantified in PSO from all the cultivars. As in most seed oil, the major plant sterol in the PSO was β-sitosterol, which ranged between 640 and 994 mg/100 g PSO. The values were higher than those reported from other fruit seed oils such as grape (231 mg/100 g) [48], and sour cherry (851 mg/100 g) [37] suggesting that PSO is a good source of phytosterols. The high concentration of β-sitosterol in the samples encourages the use of PSO as a food supplement and nutraceutical for the regulation of plasma cholesterol absorption. Stigmasterol varied from 26.0 to 52.0 (Table 2). The results on β-sitosterol and stigmasterol were consistent with those reported by Caligiani et al. [49] and Verardo et al. [50], from ethyl ether and chloroform/methanol PSO extracts, respectively.

On the other hand, the study of Fernandes et al. [51] reported lower levels of β-sitosterol and stigmasterol from petroleum ether extracted PSO. The finding that PS enzymatic pretreatment significantly enhanced stigmasterol from ‘Acco’ oil extracts by 45%, suggests that enzymatic pretreatment reduced complexation of the phytosterols with the seed polysaccharides and enhanced their mass transfer into the oil phase. Contrarily, the level of β-sitosterol significantly decreased in ‘Acco’ oil extracts by 13% after PS enzymatic pretreatment (Table 2). With respect to ‘Wonderful’ and ‘Herskawitz’ oil extracts, PS enzymatic pretreatment had no significant impact on the phytosterol content. The results suggest that the applied enzymatic pretreatment conditions did not cause a significant breakdown of the bonding forces between the phytosterols and the seed matrices in ‘Wonderful’ and ‘Herskawitz’, regardless of the notable disintegration of the seed matrices (Figure 2). This further explains that, in the seed matrices, phytosterols may exist in different forms, as either free compounds, esters of fatty acids, or glycosides, which may vary with cultivar [51]. As alluded in previous studies, this may significantly affect their dissociation from the seed matrix and release into the oil phase [52]. Insignificant effect of seed enzymatic pretreatment on the extracted oil phytosterols has also been reported in prior research. Ramadan et al. [46] reported that enzymatic pretreatment (Cellulase EC, Pectinase L 40 (1:1), 50 °C, pH: 4.3, enzyme concentration: 2% (*w*/*w*), 2 h) of goldenberry seed did not significantly affect the phytosterols content of the extracted oil.

### 2.6. Fatty Acids Composition

**Figure 6:**
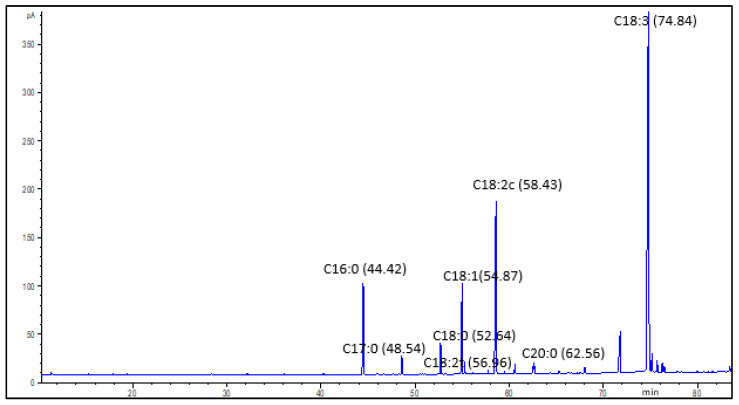
Representative gas chromatography–mass spectrometry (GC–MS) chromatograph of the fatty acids methyl esters (FAMES) from the pomegranate seed oil showing the major FAMES and their retention times. C16:0 = palmitic acid, C17:0 = heptadecanoic acid (internal standard), C18:0 = stearic acid, C18:1 = oleic acid, C18:2c = linoleic acid, C18:2t = linolenic acid, C18:3 = punicic acid, C20:0 = arachidic acid.

Figure 6 shows a representative GC chromatogram of fatty acids identified in PSO from all the cultivars. The primary fatty acids in all samples were punicic acid, which ranged between 57.2 and 67.2% and linoleic acid, which varied from 12.8 to 20.1%. Other fatty acids present in the PSO were palmitic acid (5.7–8.9%), stearic acid (2.3–2.9%), oleic acid (7.6–9.4%), linolenic acid (0.06–0.08%), and arachidic acid (0.68–0.90%) (Table 3). These results were comparable to those reported by Tian et al. [7] from the solvent, supercritical fluid, and ultrasound assisted solvent extraction of PSO. Total saturated fatty acids (SFA) ranged from 9.5 to 12.6% of total fatty acid content, monounsaturated fatty acids (MUFA) varied from 7.6 to 9.4%, and polyunsaturated fatty acids (PUFA) were the predominant class of fatty acids and accounted for 77.3 to 82.5% of the total fatty acids.

PS enzymatic pretreatment had a significant impact on PSO from ‘Wonderful’, whilst it showed an insignificant influence on ‘Herskawitz’ and ‘Acco’ oil extracts. For instance, punicic acid significantly decreased by 3.2% in ‘Wonderful’ oil extracts after PS enzymatic pretreatment. The finding that punicic acid significantly decreased after PS enzymatic pretreatment was unusual and an undesirable development since punicic acid is implicated with most of the PSO health benefits. Nevertheless, this could be linked to the significant increase in fatty acid oxidation after enzymatic pretreatment (Table 1). On the other hand, no significant effect on punicic acid from ‘Herskawitz’ and ‘Acco’ oil extracts was observed after seed enzymatic pretreatment. PS enzymatic pretreatment significantly increased palmitic acid and arachidic acid of ‘Wonderful’ oil extracts by 54 and 6%, respectively, whilst it slightly, but significantly, decreased stearic acid by 0.7%. Oleic acid (15%), linoleic acid (9%), linolenic acid (14%), SFA (33%), MUFA (15%), and PUFA (0.2%) also significantly increased in the oil extracted from ‘Wonderful’ after PS enzymatic pretreatment. However, the total unsaturated to saturated fatty acids ratio significantly decreased by 24% after enzymatic pretreatment of ‘Wonderful’ seed, which could be explained by the significant increase and decrease in SFA and PUFA, respectively. Irrespective of this, the significant increase in linoleic and linolenic acids in ‘Wonderful’ oil extracts after PS enzymatic pretreatment is an indication that the oil still had nutritional benefits. Linoleic and linolenic acids are two essential fatty acids for humans, who must obtain them from the diet and, therefore, maximum extraction of these valuable compounds from oilseeds is of utmost importance. Consumption of essential fatty acids has been associated with the prevention of certain chronic diseases, such as cardiovascular, cancer, and inflammatory diseases [53]. The result that enzymatic pretreatment of ‘Herskawitz’ and ‘Acco’ PS did not significantly affect the oil fatty acid content is consistent with prior researches. For example, Concha et al. [54] and Latif and Anwar [16] reported a lack of variation in the fatty acid content of oil extracted from rosehip and hemp seed, respectively after enzymatic pretreatment.

### 2.7. Principal Component Analysis

**Figure 7:**
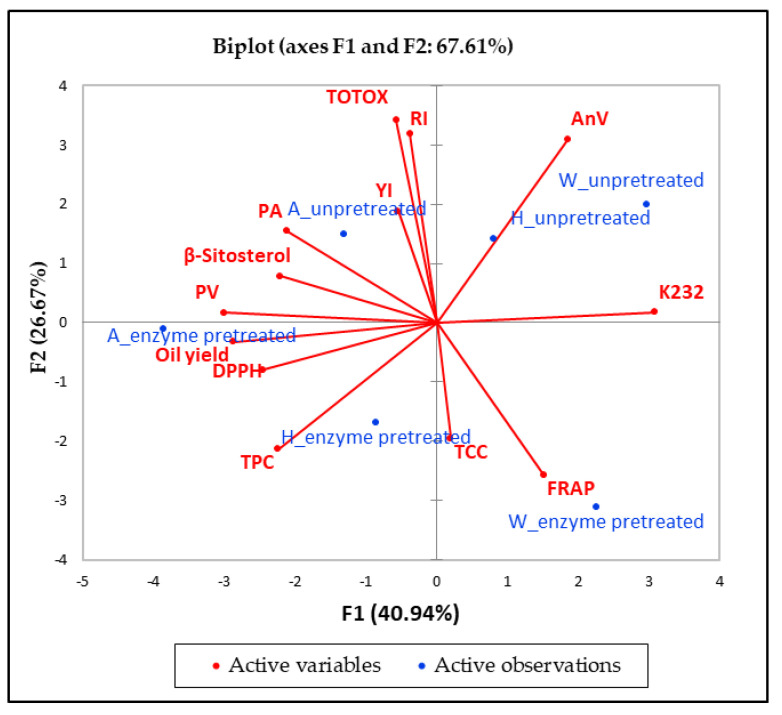
Principal component analysis data of pomegranate seed oil (PSO) quality attributes from un-pretreated and enzyme pretreated (1.7%, 40 °C, pH = 4.5 and 5 h) seeds of the studied pomegranate cultivars, YI = yellowness index, RI = refractive index, PV = peroxide value, AV = ρ-anisidine value, TOTOX = total oxidation value, K232 = conjugated dienes, TCC = total carotenoid content, TPC = total phenolic content, PA = punicic acid, DPPH = 2.2 = diphenyl = 1 = picrylhydrazyl, FRAP = ferric reducing antioxidant power, H = ‘Herskawitz’, W = ‘Wonderful’, A = ‘Acco’.

The dimension reduction property of principal component analysis (PCA) is valuable in exploring the treatments contributing more to the variation in the data. Therefore, to have a better understanding of cultivar and PS enzymatic pretreatment effect on oil quality, PCA was conducted on the multivariate data (Figure 7). The first two components (F1 and F2) had the highest eigenvalues (5.3 and 3.5, respectively) and accounted for 67.61% of the total variability, suggesting that the variation in PSO quality due to cultivar and seed enzymatic pretreatment was predominantly explained by these two principal components. Figure 7 shows that F1, which accounted for 40.94% of total variability was positively correlated with K232 (0.973), while it was negatively associated with oil yield (−0.903), PV (–0.945), TPC (−0.707), DPPH radical scavenging capacity (−0.772), punicic acid (−0.665), and β-sitosterol (−0.695). F1 was also positively correlated with ‘Wonderful’ oil extracts from un-pretreated seeds. On the other hand, it was negatively associated with ‘Acco’ oil extracts from enzyme pretreated seeds. F1 demonstrates that, in addition to increasing oil yield, enzymatic pretreatment of ‘Acco’ seed improved the oil TPC and DPPH radical scavenging capacity, which could be implicated in the decrease of conjugated dienes. The contribution of phenolic compounds to the oil DPPH radical scavenging capacity has been reported in earlier studies [55]. It is worthy to note that despite the increase in the oil antioxidant activity there were slight increases in PV. However, the values (0.04–0.33 meqO_2_/kg PSO) were lower than the limit in the Codex Alimentarius Commission (CODEX STAN 19-1981) standard for crude seed oil (15 meqO_2_/kg oil) [30]. F2, which accounted for 26.67% of the total variation, was contributed by oil extracts from untreated PS, which were significantly high in RI (0.808), AV (0.785), and TOTOX (0.869). In addition, F2 was contributed by ‘Wonderful’ and ‘Herskawitz’ oil extracts from enzyme pretreated seeds, which were associated with FRAP (−0.653). F2 illustrates that enzymatic pretreatment of PS significantly reduced oxidation of fatty acids, a phenomenon that can be explained by increased extractability of antioxidant compounds, such as the phenolics (Figure 4b). TCC and YI were poorly represented in both F1 and F2. Overall, it can be stated that enzymatic pretreatment of PS improved oil yield, bioactive compounds, and antioxidant activity, whilst reducing oil degradation.

## 3. Materials and Methods

### 3.1. Materials

Fresh good quality pomegranates (cvs. Wonderful, Herskawitz, Acco) were harvested at commercial maturity (total soluble solids: 14.02–16.61 °Brix) from Blydeverwacht farm, Wellington, (33°48′0″S, 19°53′0″E) in Western Cape Province, South Africa, between February and April, during the 2019 season. Pomegranate seeds (PS) were separated from the juice and thoroughly washed with tap water prior to oven drying at 55 ± 2 °C for 24 h and kept at 4 ± 2 °C and 92 ± 3% RH until use [56,57]. Commercial enzymes used in this study including Pectinex Ultra SPL (activity: 3800 PGNU/mL), Flavourzyme 100 L (activity: 500 LAPU/mL) and cellulase (activity: 1000 U/g) were supplied by Novozymes, Denmark and Sigma, South Africa. The enzymes were kept at 4 ± 2 °C until required.

### 3.2. Enzyme Pretreatment

Uniformly ground pomegranate seed powder (<1 mm particle size) was thermally conditioned in an oven at 80 ± 2 °C for 10 min to deactivate the endogenous enzymes [16]. Pectinex Ultra SPL, Flavourzyme 100 L, and cellulase crude enzymes were mixed in equal proportions. An enzyme suspension of 1.7% (*v*/*v*) concentration was prepared from the mixed enzymes in sodium citrate buffer at pH 4.5 and added to the samples. The enzyme suspension to seed ratio was kept constant at 900 µL/g (dry basis) in all experiments. The enzyme concentration and pH were established in a previous experiment using response surface methodology (RSM) (unpublished). The samples were hydrolysed at 40 ± 2 °C in a water bath for 5 h with continuous stirring at 150 rpm using an overhead stirrer (Scientific, Cape Town, South Africa), optimum conditions reported in prior research [58]. The hydrolysed samples were frozen at −80 °C for 24 h to deactivate the exogenous enzymes after which they were dried in a freeze dryer (VirTis Co., Gardiner, NY, USA) to approximately 4% (*w*/*w*) moisture content [46]. 

### 3.3. Oil Extraction

PSO from un-pretreated and enzyme pretreated seeds was extracted following the method described by Kaseke et al. [59]. PS (30 g) were mixed with ethanol (1: 5) in 500 mL plastic caped bottles and then sonicated at 40 ± 5 °C for 40 min using an ultrasound bath (Separation Scientific, Cape Town, South Africa) (700 W, 40 kHz, and 500 × 300 × 150 mm (internal dimensions)).The extracted oil was then filtered before solvent recovery under vacuum using a rotor vapour (G3 Heidolph, Schwabach, Germany). PS without enzyme pretreatment were used as control samples. PSO extraction yield was defined as gram per hundred-gram pomegranate seed (g/100 g seed). The triplicated (*n* = 3) PSO samples were packed in brown bottles and stored at 4 ± 2 °C for further analyses [8].

### 3.4. Pomegranate Seeds Microstructure Analysis

To evaluate the effect of enzymatic pretreatment on the morphology of pomegranate seed, untreated and enzyme treated pomegranate seed powders were subjected to scanning electron micrograph studies using a field emission scanning electron microscope (FESEM) with digital image recording (Thermo Fisher Apreo, Hillsboro, OR, USA). Specimens mounted on aluminium stubs were sputter-coated with a thin layer of gold (10 nm thick) using a gold sputter coater (EM ACE200, Leica, Wetzlar, Germany) to improve conductivity of the sample. The samples were examined at a voltage of 2 Kv.

### 3.5. Determination of PSO Quality Indices

#### 3.5.1. Yellowness and Refractive Index

Refractive index at 25 °C was measured using a calibrated Abbe 5 refractometer (Bellingham + Stanley, Kent, United Kingdom). The yellowness index (YI) was calculated from lightness (L*) and yellowness (b*) values measured using a calibrated Chroma meter CR-410 (Konica Minolta, Inc., Tokyo, Japan) [28].

(1) $$\mathrm{YI}=\frac{142.86b^{*}}{L^{*}}$$

#### 3.5.2. Peroxide Value, Conjugated Dienes, ρ-Anisidine Value, and Total Oxidation Value

PSO peroxide value (PV) was measured using the modified ferrous oxidation–xylenol orange (FOX) method described by Cruz et al. [60]. Conjugated dienes (K232) and ρ-anisidine value (AV) were determined according to ISO 3656 and AOCS. Method Cd 18-90, respectively [61,62]. The formula in Equation (2) was used to calculate total oxidation (TOTOX) value.

(2) TOTOX =2PV+AV

### 3.6. Bioactive Compounds and Antioxidant Activity Determination

#### 3.6.1. Total Carotenoids and Total Phenolic Content

The method of Ranjith et al. [63] was used to determine total carotenoid content (TCC). Briefly, absorbance of the vortexed and centrifuged (Centrifuge 5810R, Eppendorf, Germany) hexane oil extracts were measured at 460 nm using a UV spectrophotometer (Helios Omega, Thermo Scientific, Waltham, MA, USA). The absorbance of the β-carotene standard was linear between 0.5 and 100 µg/mL. The results were expressed as mg β-carotene/100 g of PSO. Total phenolic content (TPC) was determined using the Folin–Ciocalteu method [45]. In brief, the reaction mixture contained 100 µL of PSO extracts, 500 µL of the Folin–Ciocalteu reagent and 1.5 mL of 20% (*w*/*v*) sodium carbonate and 6 mL of distilled water. After incubation in the dark for 30 min, the absorbances of the samples were measured at 760 nm using a UV Spectrophotometer (Helios Omega, Thermo Scientific, Waltham, MA, USA). The results were reported as milligram gallic acid equivalent per g PSO (mg GAE/g PSO).

#### 3.6.2. Phytosterol Composition 

The gas chromatography–mass spectrometry (GC–MS) method was applied to measure phytosterol composition [51]. PSO (0.1 g) was saponified using 2.5 mL of the saponification reagent prepared from 94 mL of absolute ethanol, 6 mL of 33% (*w*/*v*) potassium hydroxide, 500 µL of 20% (*w*/*v*) ascorbic acid). An internal standard (100 µL of 5α-Cholestane (1000 mg/L) in chloroform) was added to each sample before the mixture was vortexed and incubated in an oven at 60 °C for 1 h. After cooling in ice for 10 min, distilled water (5 mL) and chloroform (2 mL) were added and the mixture vortexed and centrifuged at 3000 rpm for 4 min (Centrifuge 5810R, Eppendorf, Horsholm, Germany). The chloroform extracts (500 µL) were concentrated to ± 200 µL in 2 mL glass vials with a gentle stream of nitrogen. To 100 µL of the concentrated chloroform extracts, pyridine (100 µL), and N.O-Bis (trimethylsilyl) trifluoroacetamide (30 µL) were added. The mixture was vortexed before derivatization in an oven at 100 °C for 1 h. The GC–MS (Thermo Scientific Co. Ltd., Milan, Italy) was used to analyse the silylated sterol fractions. The samples were injected at 100 °C and temperature held for 2 min, before ramping to 250 °C at the speed of 7 °C/min and the temperature maintained for 2 min. Helium (1 mL/min) was the carrier gas and the split ratio of 5:1 and injection volume of 1.0 μL were used. The peaks were identified using a standard containing a mixture of sterols (β-sitosterol and stigmasterol). The retention times were used to identify the phytosterol compounds. The final results were reported as mg/100 g of PSO.

### 3.7. Antioxidant Activity 

#### 3.7.1. Radical Scavenging Capacity

The 2.2-diphenyl-1-picryl hydrazyl (DPPH) assay was used to measure the PSO antiradical activity [64]. PSO (100 µL) was added to 2.5 mL of 0.0004% (*w*/*v*) DPPH in 80% (*v*/*v*) methanol (freshly prepared). The vortexed samples were incubated in the dark for 60 min, after which the absorbance was measured at 517 nm using a UV spectrophotometer (Helios Omega, Thermo Scientific, Waltham, MA, USA). A negative control of DPPH in 80% methanol was applied. The standard curve (5–100 mM) was prepared using Trolox and the results were expressed as mmol Trolox/g of PSO.

#### 3.7.2. Ferric Reducing Antioxidant Power

Ferric reducing antioxidant power (FRAP) of PSO was determined by mixing 40 µL methanol oil extracts, 200 µL distilled water, and 1.8 mL FRAP reagent [65]. The FRAP reagent was prepared by mixing 2.5 mL of 10 mM 2.4.6-Tri(2-pyridyl)-s-triazine (TPTZ) solution in 40 mM HCl and 2.5 mL of 20 mM FeCl_3_ and 25 mL of 0.3 M acetate buffer, pH 3.6, and warming the mixture at 37 °C for 10 min. After incubating the samples at 37 °C for 30 min, the absorbances were measured at 593 nm using a UV spectrophotometer (Helios Omega, Thermo Scientific, Waltham, MA, USA). The results were reported as mmol Trolox/g of PSO.

### 3.8. Analysis of Fatty Acid Composition

The fatty acid methyl esters prepared according to Mphahlele et al. [66], were separated on a GC–MS system (6890N, Agilent technologies network) coupled to an Agilent technologies inert XL EI/CI Mass Selective Detector (MSD) (5975B, Agilent Technologies Inc., Palo Alto, CA, USA). Helium was employed as the carrier gas at a flow rate of 0.017 mL/s. One microlitre of sample was injected in a split ratio of 10:1. The oven temperature was programmed as follows: 100 °C/min, 180 °C at 25 °C/min and held for 3 min, 200 °C at 4 °C/min and held for 5 min, 280 °C at 8 °C/min, and 310 °C at 10 °C/min and held for 5 min. The pomegranate seed oil fatty acids profiles were identified by comparing their retention times. The relative content (%) of each fatty acid was calculated by dividing the peak area of each fatty acid by the total peak area of all the fatty acids identified.

### 3.9. Statistical Analysis

Data are presented as mean ± SD (standard deviation). Variables were compared by one-way analysis of variance (ANOVA) using Statistica software (Statistical v13, TIBC, Palo Alto, CA, USA). Significant differences between means were determined using Duncan’s multiple range test. Microsoft Excel (Version: 16.0.13029.20344, Microsoft Cooperation, Washington, DC, USA) was used for graphical presentations. The relationship between variables was determined by performing principal component analysis (PCA) using Microsoft Excel software (XLSTAT 2019.4.1.63305, Addinsoft, NY, USA).

## 4. Conclusions

In conclusion, treating pomegranate seeds with enzymes before oil extraction is an alternative way to improve the oil yield and quality. The study showed that enzymatic pretreatment of PS enhanced oil yield, TPC, TCC and DPPH radical scavenging capacity, regardless of cultivar. This is desirable to the food and nutraceutical industries, with respect to improved profits and formulation of functional foods and nutraceutical products. Enzymatic pretreatment of seeds may not affect the quality of extracted oil with respect to K232, AV, and TOTOX. Moreover, fatty acids, phytosterols, and FRAP, were not significantly affected by enzymatic pretreatment of pomegranate seeds. Compared to ‘Wonderful’ and ‘Herskawitz’, ‘Acco’ pomegranate could be considered a preferable cultivar for seed and enzymatic pretreatment to improve yield and enhance antioxidant capacity of pomegranate seed oil. In this sense, the study demonstrated that PS enzymatic pretreatment improvement of oil quality is cultivar dependent.

## Figures and Tables

**Table 1:** Physicochemical properties of un-pretreated and enzyme pretreated seeds (1.7%, 40 °C, pH = 4.5 and 5 h) from selected pomegranate cultivars.

Cultivar/Treatment						
Parameter	Wonderful		Herskawitz		Acco	
	Un-Pretreated	Enzyme Pretreated	Un-Pretreated	Enzyme Pretreated	Un-Pretreated	Enzyme Pretreated
RI	1.5177 ± 0.0000 ^a^	1.5177 ± 0.0001 ^a^	1.5177 ± 0.0000 ^a^	1.5175 ± 0.0000 ^a^	1.5176 ± 0.0000 ^a^	1.5177 ± 0.0001 ^a^
K232	0.29 ± 0.02 ^b^	0.28 ± 0.03 ^b^	0.24 ± 0.02 ^ab^	0.20 ± 0.02 ^a^	0.22 ± 0.01 ^ab^	0.17 ± 0.04 ^a^
PV	0.04 ± 0.01 ^e^	0.08 ± 0.02 ^d^	0.09 ± 0.01 ^d^	0.12 ± 0.01 ^cd^	0.22 ± 0.02 ^b^	0.33 ± 0.04 ^a^
AV	1.6 ± 0.2 ^a^	1.1 ± 0.0 ^cb^	1.4 ± 0.2 ^ac^	0.9 ± 0.0 ^c^	1.3 ± 0.1 ^ac^	1.0 ± 0.12 ^bc^
TOTOX	1.7 ± 0.2 ^a^	1.2 ± 0.1 ^ac^	1.5 ± 0.2 ^ac^	1.1 ± 0.0 ^bc^	1.8 ± 0.1 ^a^	1.7 ± 0.2 ^ab^

Means ± standard deviation of analysis (*n* = 3). Different superscript letters in the same row indicate significant difference (*p* < 0.05), according to Duncan’s multiple range test. RI = refractive index (25 °C), K232 = conjugated dienes, PV = peroxide value (meqO_2_/kg PSO), meqO_2_/kg = milliequivalents of active oxygen per kg), AV = ρ-anisidine value, TOTOX = total oxidation value.

**Table 2:** Phytosterol composition (mg/100 g PSO) of un-pretreated and enzyme pretreated seeds (1.7%, 40 °C, pH = 4.5 and 5 h) from three pomegranate cultivars.

Cultivar/Treatment						
Phytosterol	Wonderful		Herskawitz		Acco	
	**Untreated**	**Enzyme Treated**	**Untreated**	**Enzyme Treated**	**Untreated**	**Enzyme Treated**
Stigmasterol	26.0 ± 0.2 ^bc^	35.4 ± 4.9 ^b^	45.8 ± 1.5 ^a^	52.0 ± 5.4 ^a^	20.5 ± 3.1 ^c^	29.8 ± 1.5 ^bc^
β-Sitosterol	679 ± 31 ^b^	640 ± 168 ^b^	868 ± 24 ^a^	994 ± 234 ^a^	991 ± 64 ^a^	863 ± 67 ^a^

Means ± standard deviation of analysis (*n* = 3). Different superscript letters in the same row indicate significant difference (*p* < 0.05), according to Duncan’s multiple range test.

**Table 3:** Fatty acid composition of un-pretreated and enzyme pretreated seeds (1.7%, 40 °C, pH = 4.5 and 5 h) from three pomegranate cultivars.

Cultivar/Treatment						
Fatty Acid	Wonderful		Herskawitz		Acco	
	Un-Pretreated	Enzyme Pretreated	Un-Pretreated	Enzyme Pretreated	Un-Pretreated	Enzyme Pretreated
Palmitic acid	5.7 ± 0.3^c^	8.9 ± 0.2 ^a^	7.0 ± 0.2 ^bc^	7.3 ± 0.5 ^b^	7.8 ± 0.3 ^ab^	7.8 ± 0.2 ^ab^
Stearic acid	2.9 ± 0.1^c^	2.9 ± 0.2 ^a^	2.3 ± 0.0 ^bc^	2.4 ± 0.1 ^b^	2.9 ± 0.0 ^ab^	2.8 ± 0.0 ^ab^
Oleic acid	7.9 ± 0.8^c^	9.1 ± 0.1 ^a^	7.6 ± 0.1 ^bc^	7.7 ± 0.3 ^b^	9.3 ± 0.1 ^ab^	9.4 ± 0.1 ^ab^
Linoleic acid	18.3 ± 1.7 ^c^	20.1 ± 0.2 ^a^	15.2 ± 0.7 ^bc^	15.6 ± 1.4 ^b^	12.8 ± 0.6 ^ab^	14.3 ± 0.4 ^ab^
Punicic acid	59.1 ± 2.7 ^c^	57.2 ± 0.4 ^a^	67.2 ± 1.0 ^bc^	65.6 ± 2.5 ^b^	66.3 ± 1.0 ^ab^	64.9 ± 0.7 ^ab^
Linolenic acid	0.07 ± 0.01 ^c^	0.08 ± 0.01 ^a^	0.06 ± 0.00 ^bc^	0.06 ± 0.01 ^b^	0.06 ± 0.00 ^ab^	0.06 ± 0.01 ^ab^
Arachidic acid	0.85 ± 0.04 ^c^	0.90 ± 0.04 ^a^	0.68 ± 0.01 ^bc^	0.70 ± 0.04 ^b^	0.81 ± 0.03 ^ab^	0.83 ± 0.02 ^ab^
SFA	9.5 ± 0.4 ^c^	12.6 ± 0.2 ^a^	10.0 ± 0.2 ^bc^	10.5 ± 0.7 ^b^	11.5 ± 0.3 ^ab^	11.4 ± 0.3 ^ab^
MUFA	7.9 ± 0.8 ^c^	9.1 ± 0.1 ^a^	7.6 ± 0.1 ^bc^	7.7 ± 0.3 ^b^	9.3 ± 0.1 ^ab^	9.4 ± 0.1 ^ab^
PUFA	77.5 ± 1.1 ^c^	77.3 ± 0.4 ^a^	82.5 ± 0.4 ^bc^	81.3 ± 1.2 ^b^	79.2 ± 0.4 ^ab^	79.2 ± 0.4 ^ab^
UFA:SFA ratio	9.1 ± 0.4 ^c^	6.9 ± 0.1 ^a^	9.0 ± 0.2 ^b^	8.6 ± 0.5 ^b^	7.7 ± 0.2 ^b^	7.8 ± 0.2 ^b^

Means ± standard deviation of analysis (*n* = 3). Different superscript letters in the same row indicate significant difference (*p* < 0.05), according to Duncan’s multiple range test. % = relative area, SFA = saturated fatty acid, UFA = unsaturated fatty acid, MUFA = monounsaturated fatty acid, PUFA = polyunsaturated fatty acid.

## Data Availability

Not Applicable.

## Sample Availability

Samples of the compounds are available from the authors.
